# Author Correction: Quantifying the relationship between spreading depolarization and perivascular cerebrospinal fluid flow

**DOI:** 10.1038/s41598-023-47363-7

**Published:** 2023-11-15

**Authors:** Saikat Mukherjee, Mahsa Mirzaee, Jeffrey Tithof

**Affiliations:** 1 Department of Mechanical Engineering, University of Minnesota, Minneapolis, MN 55455 USA; 2 Department of Mechanical Engineering, Iowa State University, Ames, IA 50011 USA

Correction to: *Scientific Reports* 10.1038/s41598-023-38938-5, published online 31 July 2023

The original version of this Article contained an error in Reference 8, which was incorrectly given as:

“Mestre, H., Mori, Y. & Nedergaard, M. The brain’s glymphatic system: Current controversies. Trends Neurosci. 43, 458–466 (2020).”

The correct reference is listed below:

“Mestre, H., Du, T., Sweeney, A., Liu, G., Samson, A., Peng, W., Mortensen, K., Stæger, F., Bork, P., Bashford, L., Toro, E., Tithof, J., Kelley, D., Thomas, J., Hjorth, P., Martens, E., Mehta, R., Solis, O., Blinder, P., Kleinfeld, D., Hirase, H., Mori, Y., Nedergaard, M. Cerebrospinal fluid influx drives acute ischemic tissue swelling. Science 367 (2020). (https://www.science.org/doi/abs/10.1126/science.aax7171)”

The original Article has been corrected.

